# Principal components ancestry adjustment for Genetic Analysis Workshop 17 data

**DOI:** 10.1186/1753-6561-5-S9-S66

**Published:** 2011-11-29

**Authors:** Jing Jin, Jane E Cerise, Sun Jung Kang, Eun Jung Yoon, Seungtai Yoon, Nancy R Mendell, Stephen J Finch

**Affiliations:** 1 Department of Applied Mathematics and Statistics, State University of New York at Stony Brook, Stony Brook, NY 11733, USA; 2 Henri Begleiter Neurodynamics Laboratory, Department of Psychiatry and Behavioral Sciences, SUNY Downstate Medical Center, 450 Clarkson Avenue, Box 1203, Brooklyn, NY 11203, USA; 3 Seaver Autism Center and Department of Psychiatry, Mount Sinai School of Medicine, One Gustave L. Levy Place, Box 1668, New York, NY 10029, USA

## Abstract

Statistical tests on rare variant data may well have type I error rates that differ from their nominal levels. Here, we use the Genetic Analysis Workshop 17 data to estimate type I error rates and powers of three models for identifying rare variants associated with a phenotype: (1) by using the number of minor alleles, age, and smoking status as predictor variables; (2) by using the number of minor alleles, age, smoking status, and the identity of the population of the subject as predictor variables; and (3) by using the number of minor alleles, age, smoking status, and ancestry adjustment using 10 principal component scores. We studied both quantitative phenotype and a dichotomized phenotype. The model with principal component adjustment has type I error rates that are closer to the nominal level of significance of 0.05 for single-nucleotide polymorphisms (SNPs) in noncausal genes for the selected phenotype than the model directly adjusting for population. The principal component adjustment model type I error rates are also closer to the nominal level of 0.05 for noncausal SNPs located in causal genes for the phenotype. The power for causal SNPs with the principal component adjustment model is comparable to the power of the other methods. The power using the underlying quantitative phenotype is greater than the power using the dichotomized phenotype.

## Background

One limitation of genome-wide association studies is that population stratification can be a confounding variable. Population stratification occurs when there are systematic ancestry differences in allele frequencies between case subjects and control subjects. If not taken into account, population stratification can cause false-positive and/or false-negative findings [1] and can produce spurious associations [2]. Principal components analysis can be used to correct for population stratification by applying methods that infer genetic ancestry [3]. Population stratification is mainly due to the demographic history of a population, natural selection, and random fluctuations resulting from admixture. In this paper we examine the statistical properties of analysis procedures used in genome-wide association studies by adjusting principal components (PCs) across the whole genome. Another approach is to use local PC adjustment [4], but the Genetic Analysis Workshop 17 (GAW17) genotype data are not sufficiently extensive to consider this strategy.

The GAW17 data set is composed of mini-exome simulated data using 697 unrelated subjects from the 1000 Genomes Project. The quantitative phenotypes Q1 and Q2 are generated as normally distributed phenotypes. We document the *p*-value of the test of the coefficient of a genotype with and without adjusting for population stratification in selected genes known not to cause the phenotypes Q1 and Q2. We compare the power of the regression coefficient test when using PCs for ancestry adjustment with the power when using the seven populations given as ancestry controls for all the genes known to cause phenotypes Q1 and Q2. We study two types of phenotype, quantitative and dichotomized, test all the single-nucleotide polymorphisms (SNPs) that cause Q1 and Q2, and examine selected noncausal SNPs for these two traits.

## Methods

A SNP that causes a trait is one that is specified in the function used to simulate the trait [5,6]. Any other SNP is called noncausal. SNPs on chromosomes 12, 21, and 22 are used as SNPs not causing Q1. SNPs on chromosomes 21 and 22 are used as SNPs not causing Q2. Table 1 lists the distribution of the minor allele frequencies (MAFs) of the SNPs in the genes studied.

**Table 1:** Distribution of minor allele frequencies of SNPs in the genes studied

Genes	SNPs	MAF < 0.005		0.005 < MAF < 0.01		0.01 < MAF < 0.05		0.05 < MAF < 0.5		Total	
	
		Q1	Q2	Q1	Q2	Q1	Q2	Q1	Q2	Q1	Q2
Causal	Noncausal	60	100	5	9	12	15	9	15	86	139
	Causal	32	61	0	5	5	4	2	2	39	72
Noncausal	Noncausal	1,422	532	189	57	295	90	288	80	2,194	759

The Q1 causal genes are *ARNT*, *ELAVL4*, *FLT1*, *FLT4*, *HIF1A*, *HIF3A*, *KDR*, *VEGFA*, and *VEGFC*. The Q2 causal genes are *BCHE*, *GCKR*, *INSIG1*, *LPL*, *PDGFD*, *PLAT*, *RARB*, *SIRT1*, *SREBF1*, *VLDLR*, *VNN1*, *VNN3*, and *VWF*. The noncausal genes are all genes on chromosomes 12, 21, and 22 for Q1 and all genes on chromosomes 21 and 22 for Q2.

We dichotomize the quantitative measures Q1 and Q2 so that the top 25% of each of the 200 replicates is scored as affected (1) and others as unaffected (0).The independent variables in these analyses are selected from the number of minor alleles in the *i*th SNP genotype (SNP*_i_*), the participant’s age (Age) and smoking status (Smoking), six indicator variables of the populations (POP_1_, …, POP_6_), and the 10 ancestry-adjusted PC scores (GPC_1_, …, GPC_10_). We use the FamCC software [7] to calculate these 10 PCs. All 24,487 SNPs are used in the calculations.

We use the PLINK software [8] to fit three logistic regression models to assess the association between each SNP in the genes studied and the dichotomized phenotype. The *i*th SNP is considered associated with the phenotype when the permutation *p*-value of the coefficient of SNP*_i_* reported in the PLINK logistic regression analysis is less than 0.05. Because Q1 is affected by age and smoking, the models considered are the following: (1) the SNP model, in which each SNP is adjusted for age and smoking; (2) the population adjustment model, in which each SNP is adjusted for the populations, age, and smoking; and (3) the PC adjustment model, in which each SNP is adjusted for age, smoking, and ancestry adjustment PCs. The models are defined as follows:

SNP model:(1)

Population adjustment model:(2)

PC adjustment model:(3)

For the population adjustment model, only six indicators are needed to represent seven populations. The Luhya population is the reference population for the dichotomized phenotype, and the CEU population (European-descended residents of Utah) is the reference population for the quantitative phenotype. Because Q2 is not associated with either age or smoking, the covariates Age and Smoking are not used in the models for Q2. We also fit the three models to the continuous phenotypes Q1 and Q2 using PLINK. Each model is fitted to the 200 replicates provided.

## Results

The type I error rate (i.e., false-positive rate) for noncausal genes is the fraction of *p*-values from noncausal SNPs with permutation *p*-value less than 0.05. Table 2 contains the type I error rates for Q1 and Q2. The PC adjustment model has a type I error rate closer to 0.05 than the type I error rates for the SNP model and the population adjustment model. For Q2, the type I error rates are relatively close to the nominal value of 0.05 for each model.

**Table 2:** Type I error rates for Q1 and Q2 using all noncausal SNPs in noncausal genes

Model	MAF < 0.005		0.005 < MAF < 0.01		0.01 < MAF < 0.05		0.05 < MAF < 0.5		Total	
	
	D (%)	Q (%)	D (%)	Q (%)	D (%)	Q (%)	D (%)	Q (%)	D (%)	Q (%)
Q1										
SNP	9.7	6.0	9.0	9.3	17.0	21.2	20.9	31.3	12.1	11.7
Population adjustment	9.1	5.8	9.0	8.5	16.3	17.9	20.0	23.6	11.5	10.0
PC adjustment	6.6	5.7	6.3	6.4	6.8	6.9	5.7	6.7	6.5	6.0
Q2										
SNP	5.9	5.6	6.8	7.0	5.9	5.8	6.7	7.9	6.1	6.0
Population adjustment	6.1	5.6	7.7	7.1	5.9	5.6	5.6	5.7	6.2	5.7
PC adjustment	5.7	5.4	6.0	6.0	5.3	5.0	4.8	4.9	5.6	5.4

D is the dichotomized phenotype; Q is the quantitative phenotype. Noncausal SNPs for Q1 come from chromosomes 12, 21, and 22. Noncausal SNPs for Q2 come from chromosomes 21 and 22. Nominal type I error rate = 0.05, 200 replicates.

Tables 3 and 4 contain the results for Q1 and Q2 using all causal and noncausal SNPs in causal genes that determine that trait. For noncausal SNPs in causal genes for both Q1 and Q2, the PC adjustment model has permutation type I error rates that are closest to 0.05, although the type I error rates are slightly above the nominal value of 0.05. In Q1 the PC adjustment model has the lowest power for causal SNPs, possibly because of better control of the type I error rate. For Q2, where all null type I error rates are relatively close to the nominal rate of 0.05, the power for causal SNPs is roughly the same for the three models.

**Table 3:** Type I error rates and power for Q1 using all SNPs in causal genes

Model	MAF < 0.005		0.005 < MAF < 0.01		0.01 < MAF < 0.05		0.05 < MAF < 0.5		Total	
	
	D (%)	Q (%)	D (%)	Q (%)	D (%)	Q (%)	D (%)	Q (%)	D (%)	Q (%)
Noncausal SNPs										
SNP	13.5	7.8	13.8	14.4	16.9	16.7	18.4	23.6	14.5	11.1
Population adjustment	11.3	7.8	10.0	11.7	15.8	14.4	8.1	9.7	11.5	9.1
PC adjustment	5.7	6.3	5.4	9.3	6.1	4.2	7.6	8.9	5.9	6.4
Causal SNPs										
SNP	25.6	21.4	NA	NA	93.1	98.2	91.8	99.3	37.6	35.2
Population adjustment	22.6	21.7	NA	NA	91.3	97.7	85.3	98.3	34.7	35.4
PC adjustment	14.3	19.8	NA	NA	64.0	75.6	64.5	85.5	23.2	30.3

D is the dichotomized phenotype; Q is the quantitative phenotype. Nominal type I error rate = 0.05, 200 replicates.

**Table 4:** Type I error rates and power for Q2 using all SNPs in causal genes

Model	MAF < 0.005		0.005 < MAF < 0.01		0.01 < MAF < 0.05		0.05 < MAF < 0.5		Total	
	
	D (%)	Q (%)	D (%)	Q (%)	D (%)	Q (%)	D (%)	Q (%)	D (%)	Q (%)
Noncausal SNPs										
SNP	5.2	5.9	7.3	6.1	5.8	6.5	5.5	6.0	5.4	6.0
Population adjustment	6.3	5.7	11.2	7.8	6.1	5.4	4.9	5.7	6.5	5.8
PC adjustment	5.4	5.6	6.9	4.1	5.2	5.3	4.9	5.2	5.4	5.4
Causal SNPs										
SNP	11.9	13.3	30.4	31.5	39.8	45.6	55.8	80.8	16.0	18.2
Population adjustment	11.2	12.9	31.1	29.1	39.4	46.5	48.0	70.8	15.2	17.5
PC adjustment	11.2	12.7	26.5	24.5	36.5	44.4	45.3	69.8	14.6	16.9

D is the dichotomized phenotype; Q is the quantitative phenotype. Nominal type I error rate = 0.05, 200 replicates.

## Discussion

Because the disease status of interest is dichotomous in many studies, we study these dichotomized phenotypes. Chromosomes 21 and 22 have no causal SNPs for both Q1 and Q2. Therefore we define the SNPs on these two chromosomes as noncausal SNPs. Because other GAW17 participants have reported highly significant association between SNPs on chromosome 12 and Q1, we include the SNPs on chromosome 12 in our set of noncausal SNPs. For the genes reported here, the PC adjustment model has an empirical type I error rate that is apparently closer to the nominal level for SNPs in genes not causing the phenotype and for noncausal SNPs in causal genes, especially for genes determining Q1. The *p*-values for Q2 are much closer to the nominal level of 0.05 for each of the three models. This may be due to the way that the Q2 phenotype was generated. Although the PC adjustment model successfully controls the type I error rate, considering the actual population of origin does not. For noncausal SNPs in causal genes, the type I error rates in the PC adjustment model of the continuous Q1 measure are slightly higher than the nominal level in two of the four SNP strata. This may be due to the association between the noncausal SNPs and causal SNPs within the gene resulting from linkage disequilibrium. It may also result from multiple testing.

The power of the PC adjustment model is relatively strong and increases as the MAF increases, as expected. The power of regression modeling for the quantitative phenotype is greater than the power of logistic regression modeling of the dichotomized phenotype for both Q1 and Q2.

In this study, we compare the PC adjustment model with a model including population of origin as a factor. The PC adjustment model has both a type I error rate closer to the nominal level of 0.05 and high power. This is because, in general, PCs calculated using all SNPs contain more information about demographic history, natural selection, and random fluctuation in admixture than the population to which a participant is assigned. That is, participants’ genes may still hold genetic information that distinguishes them from the population from which they originated.

The data used here were simulated rather than real. We set our significance level to 0.05 because the number of replicates is 200. As a result, the expected number of null rejections is 10, which allows for meaningful statistical comparison. We also studied a nominal significance level of 0.01 (data not shown) and found similar control of the type I error rate except for SNPs with MAF < 0.005, where the type I error rate was 0.036, somewhat higher than expected. We could not study the type I error rate using typical genome-wide significance levels, such as 10^−8^.

## Conclusions

The PC adjustment model with permutation *p*-value controls the type I error rate in the GAW17 Q1 and Q2 phenotypes. The power of the regression analysis of the quantitative phenotype is greater than the power of the analysis of the dichotomized phenotype. There is a slight decrease in power for the PC adjustment model even when MAF < 0.005.

## Competing interests

The authors declare that there are no competing interests.

## Authors’ contributions

JJ carried out all analyses of the dichotomized trait, participated in the analysis of the quantitative trait, and drafted the manuscript. JEC carried out the analyses of the quantitative trait. EJY and SY participated in generating and analyzing the dichotomized phenotype. SJK conceived of the study and participated in its design. NRM and SJF participated in the design, coordinated the study, and helped draft the manuscript. All authors read and approved the final manuscript.
